# Transition Trajectories: Contexts, Difficulties and Consequences Reported by Young Transgender and Non-Binary Spaniards

**DOI:** 10.3390/ijerph17186859

**Published:** 2020-09-19

**Authors:** Mariana Magalhães, Marta E. Aparicio-García, Isidro García-Nieto

**Affiliations:** 1 Work, Social and Differential Psychology Department, Faculty of Psychology, Complutense University of Madrid, Campus de Somosaguas, s/n, 28223 Madrid, Spain; m.maia.magalhaes@gmail.com; 2 LGBTI Service of Madrid Community; C/ Alcalá, 22, 5° dcha, 28014 Madrid, Spain; isidrogarcianieto@gmail.com

**Keywords:** transgender, non-binary gender identity, gender nonconforming, transitioning, gender affirming care, minority stress

## Abstract

The transition process can have a significant impact on young transgender and non-binary individuals (TNBI), especially regarding their mental health. As such, this study aimed to explore the encountered difficulties and expectations of 225 young adults, between 14 and 25 years old, who identify themselves as TNBI. Four different aspects were analyzed: difficulties related to the transition process; main current difficulties; if the current difficulties are due to the participants’ sexual orientation or gender identity; and if participants feel the need of changing something in their lives. Several obstacles either before, during or after the transition process were identified; and these obstacles have serious consequences in TBNI’s mental health. Questions regarding legislation, discrimination, transphobia, lack of social support, the pathologization of transgenderism, low self-esteem, anxiety symptomatology, among others, were pointed out by the participants. The narratives collected and analyzed in the present study appear as important inputs to the literature, which can be translated into the basis of further investigations and the improvement of professional practices in the health field.

## 1. Introduction

Some individuals may not fit into the gender assigned at birth and/or not conform to the binary system of gender. The concept transgender includes a variety of nonconforming gender expressions or behaviours, although it is broadly used to refer to people whose gender identity is not fully aligned with their sex assigned at birth [1]. Some people could need a transition process that requires different steps, including changing their name, pronouns, and clothing, or hormonal interventions, among others [2]. Nevertheless, it is crucial to understand that not everyone choses to transition or to follow every possible step. In Spain, Legal Gender Recognition (LGB) was publicly regulated through the Law 3/2007, which rectified the mean of people’s sex and established the elementary requirements for civil registration for LGB (making possible changing name and gender on official documents without a mandatory requirement of genital surgery nor forced sterilization). Nevertheless, it is still compulsory to be at the legal age, have a psychiatric diagnosis and have experienced two years of certified medical treatment (even if not all transgender individuals feel the need or desire to do it).

Even though laws are evolving towards inclusion, tolerance, and recognition of transgender rights, it is important to recognize that in many countries (Spain included), prejudice and discrimination are present in the everyday life of many transgender and non-binary individuals (TNBI). Transphobia, as a form of violence directed to people who transgress gender norms, is one of the biggest challenges faced [3,4]. It can be expressed in more severe forms, as hate crimes, and less severe forms, as unwillingness to accept transgender identities by, for example, refusing to use the correct pronoun or chosen name or expecting someone to naturally identify himself/herself in terms of gender with the genitalia the person was born with. Another important barrier to be mentioned is the pathologization of transgenderism, with many (including healthcare providers) believing it is an illness and/or that every transgender individual experience anatomical dysphoria. This could be related to the gender binarism to which these individuals are exposed, making non-binary identities less valid and accepted [4].

Not rarely, these negative attitudes and violence are internalized, making one’s self-perception very negative [3]. Facing this social challenge frequently can make transgender people have a higher probability of feeling unfortunate and isolated (which may be due to suppressing transgender feelings for a long time), developing mental health disorders, and reporting suicidal ideation [5].

While discussing the influence and the impact of society among these individuals’ development, it is crucial to take into consideration the Minority Stress Model [6]. It claims that minorities (and, in specific, sexual minorities) experience unique stress processes due to their minority status (e.g., discrimination, identity concealment, internalized stigma, expectations of rejection) and, therefore, these population experience likewise increased mental health problems that will impact their lives. As seen above, transgender youth is, according to this model, a victim of these unique stress processes during their development. As such, social support (from family and friends) is crucial so this population can cope with the discrimination they face daily [7].

Transgender people experience different challenges when choosing whether to live according to their gender identity or not. Some transgender people decide to make a physical transformation to adapt their body to their gender identity. Some transgender individuals need both hormonal therapy and surgeries, while others only need some of these options and others none [8,9]. Some researches indicate that people who carry out the transition process according to their own have a safer self-image have lower depression rates and better communication in their relationships [10]. However, it has also been found that some transgender individuals become obsessed with cosmetic procedures related to discomfort with their general body image, internalized transphobia, or feelings of not being conventionally feminine or masculine [8]. Anxiety and depression are frequently related to the transition process. Additionally, many social challenges may be faced at different spheres of a person’s life: at home, with family, that may not be informed, understand and accept one’s gender identity; at the office, where the transition process demands a coming out and where one may face discrimination. Moreover, feelings of guilt may be expressed regarding personal relationships’ disruption due to disclosure regarding one’s gender identity or desire of initiating the transition process [2,10]. At this point, it is important to reflect about the importance of social support overall but especially when one is transitioning.

One of the key variables pointed out in these transition processes is the acceptance by the families. Social support during the transition process seems to be extremely important [4,7] and several benefits are identified by transgender individuals due to having a supportive family, relating to other transgender people, and feeling community connectedness [4]. Some follow-up studies conducted with children diagnosed with gender dysphoria showed that they had high levels of internalized psychopathology [11,12]. However, it has been reported that the diagnosis of gender dysphoria and its psychopathologization in a mental health context could be the cause of these higher levels of discomfort. More recent studies with children whose families support them and allow their social transition in the pre-pubertal stage have shown that these children have better mental health, with similar indicators in depression and anxiety as cisgender children [13,14]. It has also been observed that transgender children who have made the transition have significantly lower levels of internalized psychopathology than those previously found in children diagnosed with gender dysphoria (TIG) and who lived according to their gender of assignment [14]. Accordingly, a study conducted with 20 transgender men and women in adulthood showed that participants’ psychological well-being improved since the transition [7]. Other authors in a longitudinal study with 55 transgender people (22 women and 33 men) who had been hormonally blocked as teenagers found that the anxiety rates, emotional distress, and concern for their own body image were similar or better than those of the cisgender population of the same age and none of the people regretted the process followed [15]. Nevertheless, those transgender individuals who do not receive support from their relatives are eight times more likely to have attempted suicide than the rest of the population of the same age, six times more likely to have high levels of depression, and three times more likely to consume drugs. Additionally, they present a risk three times higher of becoming infected with HIV and contracting sexually transmitted diseases [16]. Families can and must play a very important role with regard to the transition process [17]. When one’s gender identity has to be kept hidden due to lack of acceptance and support by one’s family, social isolation is promoted, namely until adulthood (or the moment one finally feels comfortable to come out, or even when finally one is capable to perform the transition process autonomously). In addition, as described by the participants in this study, being involved in a transition process at an early stage (before adulthood) without family support is extremely inappropriate. The depathologization of transgenderism is, in this sense, a highly necessary process, which can prevent a negative impact on the mental health of this population whether this is due to the discrimination caused by pathologization and disinformation, or whether this is due to having to live in a body with which one does not identify with [17].

Gender transitioning is a process that calls not only for social support but also psychological and medical. As such, it is important to mention that the medical community plays a crucial role in this process, as reliable professionals are essential to ensure a safe and informed transition path [17], and should focus on creating protocols for the transition, making sure this population has their needs met. It should not be forgotten that the most significant thing is to let individuals express themselves spontaneously, providing them support and acceptance, regardless of their gender identity and its evolution [2]. However, this does not always happen. In a recent research, among the 49 participants who sought endocrine care for their transition process, 42% reported having difficulty accessing an endocrinologist with trans-specific knowledge, while 39% affirmed having difficulties accessing a supportive one [18]. Additionally, among the 44 participants who searched for a surgeon for transition-related care, one third reported difficulties accessing one with trans-specific knowledge. In the same study, finding a knowledgeable primary care physician was easier; however, finding one that was perceived to be supportive of transgender people was described as a difficult task by 53% of the participants [18].

Despite the great amount of studies analysing transgender people’s mental health, few studies focus on the difficulties they encounter during the transition process and their expectations about it. The aim of this qualitative study is to analyse how this process is experienced by transgender people, the difficulties above mentioned, if transgender people believe their sexual orientation and/or gender identity are the cause of such difficulties and what they would desire to change in their lives if they could.

## 2. Materials and Methods

### 2.1. Participants

The final sample of this study was comprised by 219 young people between 14 and 25 years old. The mean age of participants was 19.46 years (SD = 3.254). 117 identified as transgender male (people who were assigned female at birth and that self-identify as male; 53.4%), 38 as transgender women (people who were assigned male at birth and that self-identify as female; 17.4%), and 59 as non-binary (26.9%), with 5 missing responses to this question (2.3%). All participants were born in Spain. Other demographic characteristics in Table 1.

### 2.2. Procedure

The four questions under analysis were integrated in the context of a larger research on the well-being of transgender and non-binary youth [5]. Participants were selected through recruitment in Facebook, Twitter, and different associations (Daniela Foundation, FELGTB, and COGAM) in Spain, who were previously contacted in order to explain the aims of the current research and its method. The link of the online survey was sent to all of those who agreed to participate, as well as shared in the organization’s website and social media.

The four variables under analysis were inserted in a larger questionnaire. However, from the total sample (*n* = 856), only participants who met inclusion criteria (being transgender and/or non-binary; being over 18 years-old) could access the questions mentioned above, depending on previous answers. Hence, 225 participants were considered. However, 29 individuals just answered the demographic questions and for that reason were excluded from our sample. Likewise, 7 entries were found to be from the same participant, so they were excluded also.

In the online survey, participants could read a brief instruction which described the research, as well as the inclusion criteria for participating, where they had to agree to participate before answering any questions. Participation in the research was anonymous and voluntary. All performed procedures in this research were in accordance with the ethical standards of the institutional and/or national research committee and with the 1964 Helsinki declaration and its later amendments or comparable ethical standards, as well as with the position statement developed at the 2nd World Conference on Research Integrity, 2010, Singapore.

### 2.3. Instruments

A survey including questions about sociodemographic information and the life experiences regarding transgender and non-binary people was designed ad hoc for this study.

The sociodemographic information assessed in the first part of the survey contained items about gender, age, residence, and religion. The gender identity of the participants was measured by the question, “Do you consider yourself?” (with response options of “male”, “female”, “transman”, “transwoman”, or “non-binary”). Considering the answers to the previous questions, only transgender or non-binary individuals could keep answering the questionnaire. As such, two different groups were created: transgender (transman and transwoman) or non-binary. The area of residence was assessed through the question “Would you describe the area in which you live?” (with response options of “rural” or “urban”). The participants’ religion was measured by the question “What is your religion?” (with the response options “Catholic”, “Jewish”, “Muslim”, “Evangelist”, “Other”, “Atheist”, “Agnostic”, or “I prefer not to answer”).

To assess questions about the transition process, and how gender identity and sexual orientation influences the lives of our participants, four open-ended survey items were asked: “In your own words, what was your main difficulty when you were transitioning?” (Q1); “What is the main difficulty that you are facing nowadays?” (Q2); “Do you think that the cause of this difficulty is your sexual orientation or your gender identity?” (Q3); and “In your own words, if you were capable of changing something right now, what would it be and why?” (Q4).

### 2.4. Data Analysis

To describe the sample’s demographic variables, the program IBM SPSS, version 25 (IBM, Madrid, Spain), was used.

On the other hand, to analyse the answers given by our participants about the transition process, the computer-assisted qualitative data analysis software ATLAS.ti, version 8 (Atlas, Madrid, Spain), was used. In order to analyse the extracted data, participants’ answers were organized from the most recent to the older answers. Each researcher analysed the collected data individually and independently, in order to summarize the core ideas—codes—concerning each of the four different questions. These codes were discussed among the researchers, reaching for reliability and in order to reach a consensus on of them should be used throughout the analysis. Subsequently, the team carried out a cross analysis in order to group similar codes into categories, while a code could only belong to one category. As with codes, the different categories created were also discussed within a research team, having been validated by all researchers. Finally, all categories were reviewed and discussed extensively, until all researchers agreed that all relevant themes were represented.

## 3. Results

The results of this study showed the main difficulties identified by participants regarding their transition process, the ones they were facing at the time of the questionnaire and what they would change if they had the power to. Moreover, their perception of the role of their gender identity and sexual orientation in these difficulties is accessed.

### 3.1. Main Difficulty While Transitioning

Regarding the question “In your own words, what was your main difficulty when you were transitioning?”, several difficulties were mentioned. There were 27 data missing, 9 participants referred that the question did not apply to them since they had not done the transition and 6 individuals affirmed not having any difficulties while transitioning (Table 2).

Different individual-related difficulties were mentioned. Age (more particularly, being under 18 years-old) was mentioned as a significant barrier (5 participants), as well as having lack of financial resources (7). Additionally, a participant mentioned the area of living as a difficulty towards transitioning, without explaining why. Doubts about one’s sexual identity (5)—“(…) myself, knowing what I wanted (…)” (sic)—and lack of self-acceptance (5)—“(…) my self-acceptance process was also very hard (…)” (sic)—appear equally as substantial difficulties.

Family-related difficulties were an important theme that arouse, as expected due to reviewed literature. Lack of familial acceptance and support (41)—“(…) the disagreement of my parents (...) since they have never supported me or helped me start my [transition] process (…)” (sic)—together with family rejection (4)—“(…) my parents threw me out of the house (…)” (sic)—are important barriers towards the transition process, assuming a crucial role. Several misconducts are described, like refusing to use chosen pronouns and name, not allowing physical changes, among others. In fact, a participant mentioned “(…) the main difficulty is in my house, since my mom is completely against [the transition process] (…)” (sic).

Process-related difficulties were also mentioned, being mainly divided in two different categories: medical-related difficulties and law-related difficulties. Concerning law-related difficulties, participants referred law itself (3), changing to official documents (as not being able to do it or finding many obstacles to complete the procedure; 5) and bureaucratic procedures they have to go through in order to transition (2). Regarding medical-related difficulties, uninformed (5) or non-collaborative (2) medical professionals—“(…) the underrepresentation and knowledge of doctors and other health professionals of the gender spectrum outside the male/female binary (…)” (sic)—and difficulties accessing hormonal treatment (1) were stated. Additionally to both these categories, the lack of (either legal or medical) information about the transition about the process itself (11), beginning the transition process (3), the slowness of the process and waiting time for something that is “(…) hard to wait for (…)” (sic) are mentioned as main difficulties that, unfortunately, are out of the individual’s control (9).

Another main theme emerged, based on Gender Minority Stress related difficulties. Among them, participants referred the imposed traditional gender dichotomy (5) and consequent trans and nonbinary population’s invisibility (4)—“(…) people with a nonbinary gender identity have even less visibility (…)” (sic)—lack of social acceptance (7), social rejection (10) and discrimination (6)—“(…) I would disgust the world and they would not accept me (…)” (sic). When stigma is internalized by the individual, according to two participants, internalized transphobia becomes a barrier to the transition process, as it “(…)makes me hate myself and constantly doubt myself, who I am, what I identify with and how I feel (…)” (sic). As we can conclude, society plays an important role and is frequently cited as part of the difficulty or even as the main difficulty itself (8). Additionally, the attitudes from others regarding transgenderism (8) should equally be taken into account, as the way others think and perceive this phenomenon influence the individual—“(…) how I was going to deal with people, if it was going to go well or not, if it was going to be difficult overall (…)” (sic). Thus, leaving in a society that exposes an individual to such stigmatization can make it more difficult for one to come out as a transgender (22), not only to his/her family and friends, but also in general—“(…) I found it hard to have to tell it to my family and friends (…)” (sic).

Other difficulties were likewise mentioned by participants: everything being a difficulty (1); not having many (and not specifying what; 1); finding a job (1); fear (not specified; 9) and psychological symptoms (4), as anxiety, solitude, depressive symptoms, and lack of concentration.

### 3.2. Main Current Difficulty

Concerning the question “What is the main difficulty that you are facing nowadays?”, there were acknowledged 9 data missing and six participants reported not having any difficulties at the time (Table 3).

Gender-identity related difficulties are mentioned. Fear that everything is just a phase (2), being indecisive about their own identity (5) and sexual orientation (2) are described as important difficulties. Related to gender-identity, going to the bathroom (3) is reported as a difficulty. Using either public bathrooms, or more precisely the ones in educational establishments, as well as locker rooms, are a great difficulty faced, as participants feel observed and uncomfortable.

The Transition Process was once more mentioned as a difficulty, giving rise to another theme. While the process itself is mentioned by some as the main difficulty (6), other participants report taking the first step or beginning parts of the transition (8), making alterations to official documents (15) and uninformed professionals (1). Moreover, the length and duration of the transition process itself (4) was identified as an obstacle, as well as not having the availability for such a time consuming and demanding process (2). Furthermore, financial problems (15) are cited as important difficulty for both the transition process itself and completing the process in private practices (described as appealing due to the shorter waiting time).

Some of the difficulties presented were apparently related to sociodemographic characteristics and everyday life factors. Participants report as barriers age (3), or more precisely being under 18; religious beliefs (3), which can play an important part in the isolation of this population, “(…) they don’t accept me in the catholic church because I’m transsexual (…)” (P141), participants’ studies (15), and losing weight (1).

Additionally, intimate relationships and the professional context are seen as daunting. Intimate partner relationships can be challenging for transgender individuals, as finding a partner (5) is described as “(…) very difficult (…)” (P130), as well as engaging in an intimate partner relationship (1).

Job related issues were equally reported. Finding a job (8) is not easy for this population and, when employed, initiating the transition process can be difficult, either for lack of time or for fear of discrimination in the workplace. Additionally, one might fear the future work situation, after the transition. Therefore, the present and future work situation (7) was identified as a main difficulty.

Gender Minority Stressors have an important role in the everyday life of this population and, therefore, some of the named biggest difficulties are associated with them. Other people’s opinion (1), lack of acceptance (20) of these individuals—“(…) non-binary transgender people are still not accepted today (…)” (sic)—consequent discrimination (10) and further isolation (2) are frequently mentioned, while participants seem to desire to “(…) to be able to go out as I want without receiving insults or funny looks (…)” (sic). Besides lacking social integration (1) in general, a participant identified integration in the LGBTI community as an important barrier. Thus, fear of rejection or facing it (6); perceiving their invisibility and the lack of knowledge about what the non-binary is (9—“(…) it seems like there is no other option besides man/woman (…)” (sic)—which forces individuals to continually justify their gender (2); and feeling socially pressured to relate to cisnormativity (2) are reported as difficulty. Additionally, some individuals mentioned being exposed to the phenomenon of misgendering (11), “(…) the majority of people with whom they interact frequently, both friends and family, people I known, etc. recognize myself as my sex of birth and it bothers me because I feel and express myself most of all as the opposite (…)” (sic).

Psychological Symptoms and/or Disorders emerge as a theme. The most reported disorder was gender dysphoria (17), with participants reporting that “(…) I don’t feel like myself, I don’t identify myself with my body image and how others perceive me (…)” (sic). Depression was also commonly referred (11), followed by anxiety (9). Eating disorders (1), self-esteem issues (1), solitude (1), and lack of emotional control (1) were equally named as current difficulties with a significant impact on the individual.

As several mentioned difficulties were also reported in the first analysed question by different participants, it can be concluded that these are significant barriers to individuals who pretend to make the transition or are already doing it, that prevail over time.

### 3.3. Cause of the Main Current Difficulty

Regarding the question “Do you think that the cause of this difficulty is your sexual orientation or your gender identity?”, the three main themes were defined: sexual orientation or/and gender identity as causes of the biggest difficulty (138); sexual orientation or/and gender identity as unrelated factors to the biggest difficulty (138); and uncertainty concerning these causes (8). There were identified 13 data missing, and one participant replied that this question was previously responded (Table 4).

Concerning the first theme, gender identity (60) was found to be the cause of the biggest difficulty way more frequently than sexual orientation (2), which was considered an issue easier to deal with, “(…) My sexual orientation, I think I always had it figured out, that did not cause me any problem (…) nevertheless, my gender identity has made several aspects of my everyday life difficult for me (…)” (P18). Still, both were occasionally mentioned as equally responsible for this difficulty (13), with one participant mentioning that being non normative could originate uncomfortable situations, “(…) people always use me to exemplify the non-normative (…) sometimes I feel very exposed (…)” (P16). Moreover, some considered that the biggest difficulty could be partially due to sexual orientation and/or sexual identity (13).

As to the last theme, several causes of the biggest difficulty were mentioned. A sub-theme was identified—“Gender Minority Stressors”—and contained several codes related, as the society (frequently described as discriminative; 6), transphobia (1), discrimination (1), rejection (1), invisibility (1), and others being uniformed about the non-binary (1). Lack of acceptance (6) and determination (1), the pathologization of the non-normative (1) and body image (1) were other motives mentioned.

### 3.4. Possibility of Change

Regarding the question “In your own words, if you were capable of changing something right now, what would it be and why?”, 23 data missing were identified and one participant chose not to answer, 11 said they would not change anything and 2 said they would change everything (Table 5).

Different personal aspects seem to have a crucial impact in this population and, as such, several participants express they wish they could change them in order to make their lives easier. As seen in previous questions, the age (3) and being under 18 can be a significant barrier, so it only makes sense this would be something one wants to change. Likewise, the place where participants lived (6), “(…) so I can have a fresh start (…)” and fear (from both the transition process and social situations; 3) were aspects participants aimed to change. Finally, one participant describes a disconnection between her body and her emotional dimension that wishes she could change. Lastly, one participant wished he was never born, which can reflect the extreme difficulties this population faces in the most diverse areas of their lives and the incredibly significantly negative impact it can have on them.

Several participants reported aiming to change some biological aspects. Among them, are the voice (3), the physical appearance (24), the genitals (5), having breasts or having a mastectomy (7), and changing the sex itself (7).

Social aspects were equally mentioned as aspects they wanted to change. Familial acceptation and support (16) were crucial factors to the participants, who crave for more support, “(…) I would open my parents mind so they would be capable of being at least informed and wouldn’t reject redundantly something they don’t know (…)”. Therefore, participants wished their families were more informed about their situation, as well as more involved, and seek more tolerance and support from them. Friendships (4) seem to be another important topic, as participants wished they had new friendships with people that “(…) understand how I feel (…)” or the quality of their friendships was better. Furthermore, suffering bullying (1); LGBT community and services (1), which are seen as “(…) too binary (woman/man) (…) it seems they are ‘LGB’ instead of ‘LGBT’ (…)” (P185); not having an intimate partner (1) and a support network (1) are also cited as things these individuals would change if possible.

Financial aspects are commonly referred. Their economic situation (9) is something the participants would change, as they feel that having more money could help them in some way. Related to this, participants crave their financial independence (8), including in order to allow them to “(…) have more freedom regarding my identity (…)” (P48).

Gender Minority Stress, once again, plays an important part. Coming out of the closet (10) is an aspect that participants would change in different ways: while the majority wished they would have done it sooner (8), one reports that he wish they could do it (1) and one would not have done it even at all (1). Society (12) seems to be a key factor for a lot of our participants, often described as binary. It is mentioned the present “(…) chauvinist, homophobic and transphobic culture (…)” (P7), as well as the population’s lack of education and social awareness about transgenderism. However, participants wished they could live in a more inclusive society or in an “(…) utopian world, that the whole issue of gender was normalized and more inclusive... it would be the ideal solution... (…)” (sic). The faced inequalities (1) are also mentioned as something worth changing. As such, participants wished for more respect and for a change in mentalities.

It is cited that, if ever given the power of change, some aspects related to the transition process would suffer some alterations. First, changing the official documents so they match their owner’s gender identity (8) seems to be very significant as, like observed in previous questions, it is frequently mentioned. Starting and/or facilitating the access to hormonal treatment (6), as well as nobody knowing one’s sex at birth (1), are also cited as an important change.

Some gender-related aspects are referred by participants. The wish of being able to change or choosing between genders (5) is referred, which could be intrinsically related to the wish of other participants about changing their sex (above cited). Moreover, some affirm they would like to have been born not transgender but cisgender (21).

## 4. Discussion

The process of transitioning is complex and has several difficulties related to it, which could appear before, during, and after this process. As mentioned above, the Law 3/2007 regulates that only people above the legal age can perform the transition process and this is identified as a significant barrier by several participants. Being aware of their identity and not being able to do anything regarding the physical (e.g., hormonal treatments) or social changes (e.g., changing official documents) they need to become the person they know they are can be extremely harmful. As reported in several studies above [13,14,15], individuals who start some parts of the transition process present better mental health indices than those who do not or similar levels comparing to cisgender individuals. In this sense, this result reinforces the need to review legislative, medical, and social practices (among others) in order to ensure the well-being of this population and, ultimately, their mental health. Furthermore, it is emphasized that respect for the person’s right to self-determination prevents situations of victimization and psychologically and socially harmful impacts on them.

The pathologization of transgenderism is still a reality which has a negative influence in this population’s lives. Pathologization can come even from medical professionals [4]. It can be represented in different ways, one of them being believing in the myth that every transgender individual must experience anatomical dysphoria or that a non-binary identity is not as valid as a binary one [4]. Besides, as reported by several authors, the pathologization of transgenderism can lead to higher levels of psychological discomfort [13,14]. Moreover, when the problem is not having a doctor who pathologizes one’s gender identity, uninformed professionals about trans-specific knowledge can be a significant barrier [18]. When this happens, especially with the professional who was supposed to keep one safe during the transition process and prevent one’s isolation [17], the care’s quality is almost automatically harmed. As such, there is an urgent need for greater investment in the training of all professionals (medical or not) who are in contact with this population, in order to avoid episodes of discrimination. In addition, in the medical context in particular, it is important to highlight the importance of training professionals, both in terms of the possible needs of this population and at the human level. Knowing what steps to take to help a transgender or non-binary person achieve their transition goals can be framed as important as knowing how to behave in a non-discriminatory way in the context of consultation and respect their right self-determination.

Unwanted attention and discrimination are frequently cited by our participants as difficulties they would like to change, if they could. This translates the role of society, stigma, and preconceptions socially constructed that, in many ways, makes the every-day life of these individuals worse. It is worth mentioning that the discrimination this population faces often leads them to isolation and puts them at higher risk of developing mental health disorders and reporting suicidal ideation [5,19], as well as internalizing this stigma and, consequently, lowering their self-esteem [3]. These facts are equally coherent with our participants’ narratives. Moreover, as a violent conduct exclusively directed to individuals who somehow do not present themselves accordingly to gender norms, transphobia is seen by our participants as an significant barrier to their quality of life, which is coherent with previous research [3,4]. They mention not only the more visible and obvious forms (e.g., insults), but also the less visible forms (e.g., not using the correct pronoun or chosen name). Therefore, the results of this study, in line with many other investigations, point out the importance of working at a societal level in order to eliminate discrimination and violence in the face of several minorities (namely sexual minorities), as they function as social mechanisms to maintain the social order and, consequently, its systems of oppression. Besides making them live in permanent fear and promoting isolation, the aggressions this population suffer occur in the different dimensions of their lives (family, work, etc.), clearly harming them and preventing them from being themselves.

Mental health is likewise a crucial issue to be aware of when dealing with this population and their transition process. Besides the consequences of discrimination, transphobia, and internalized stigma, anxiety and depression are frequently mentioned by our participants and by them related to the transition process, which is congruent with the literature [3]. As such, the collected data may be aligned with the Minority Stress Model [6], since participants frequently report discrimination, identity concealment and rejection based on their minority identity. Therefore, this model should be taken into account when analysing the development of transgender youth and designing intervention programs for this population, as well as for their families.

People who chose transitioning are specially in need of social support, as the process is complex [7,17]. Several participants refer the lack of support they feel from their family and how they wished they could understand who they are and what they are going through. Not having family support can express itself in being at higher risk of several negative impacts (e.g., suicide attempts, consume drugs) [16]. However, the family is not the only support system a transgender individual needs, as several of them also mentioned they wish they had more friends, more supportive ones, or even a romantic partner. The wish of a closer connection to the transgender community was also cited. These bounds are likewise extremely important, as reported in the literature, bringing to these individuals several benefits [4].

Physical appearance and self-perception seem to be preponderant in the well-being of this population, being mentioned throughout the four analyzed questions. The fact that several participants name aspects related to physical appearance as something they would like to change may be an indicator of its importance. In this sense, the way a person feels about his/her appearance can influence his behavior in the social sphere of his/her life, “(…) the thing that upsets me the most at this time is to identify myself as male before everyone but, having not started with the hormone therapy yet, having an entirely female body and feeling insecure when talking to people and meeting new people through it fear that they continue to see me as a woman (…)” (sic), and even be related to social anxiety (sometimes reported by our participants). Furthermore, negative feelings are often related to physical appearance, “(…) I would change my voice and the look of my face to be able to “pass” and not feel so uncomfortable (…)” (sic), and identified as a cause of distress, which is in line with previous literature [3]. Additionally, several report that they would change the fact that they were born transgender, which can be associated not only with social aspects (e.g., transphobia) but also with one’s physical appearance and consequent congruence with the gender one identifies with.

The transition process, as mentioned by several participants, is a long one. Perhaps for this reason, we can see from the answers given to this study’s survey that several participants are at different stages of the transition process they chose to do (e.g., while participants there are who have already started hormone therapy, others have not started it yet; some mention having already come out to their loved ones, while others fear this part of the process). Nevertheless, it is possible to conclude through the analysis that there are several difficulties faced by this population that are identified by the majority of the participants: sometimes, as the greatest current difficulty and other times as the greatest difficulty during the transition process. Although, for some, these two time periods may coincide, for others they do not. Thus, it is possible to conclude that some of the difficulties mentioned are constant over time (e.g., lack of social acceptance, levels of anxiety, depression) and may even be related to each other. For example, discrimination is frequently mentioned throughout the different answers to all the four questions, which is in line with what was reported by Verbeek and colleagues (2020) [7], that argued that stigmatization and discrimination remain after transitioning. In this sense, it is important to create awareness about the harmful impact that these difficulties can have on the individual, in the long and short term. In fact, it is even possible to argue that some of the difficulties mentioned by the participants, such as anxiety and depression, may already be, actually, consequences of the discriminative society that we live in and that, in different ways, mistreats this population, which would be in line with previous literature [6]. As a participant reports, sometimes the main difficulty is the “fear of what lies ahead”.

## 5. Conclusions

The present study highlights different aspects of the transitional process, namely several difficulties that can truly harm one’s development, helping to advance insight regarding the transition process through the eyes of transgender and non-binary youth in the Spanish context. As mentioned, these difficulties can be related to different dimensions of one’s life and have a significant impact.

The main difficulties that our participants point out are related to difficulties they have in their transition process, lack of family support, and medical services. Improving these support systems seems to be fundamental to improving the quality of life of transgender people and facilitating their transition processes, if desired. Therefore, these results that arise from participants’ narratives should be considered when analysing and originating intervention targeting this population. It would also be important to build other social models that do not necessarily involve directing them into a transition process if this is not desired.

Some limitations regarding this study should be addressed. In order to improve the generalization of results, the sample should be expanded. Additionally, the results could have been biased, as the sample was formed throughout the collaboration of several LGTB Spanish associations and people who are not involved with them or that have a limited access to the internet could have been left out. Therefore, the replication of this study with a broader sample should be considered. Secondly, there is a large amount of missing data, so this study should be replicated with personal interviews that will facilitate clarifying why they did not want to answer or even that they did answer in the context of an interview. Thirdly, these results were collected through open-ended questions in an on-line questionnaire, with limited space for response. As such, future research should explore the same topics through interviews or focus group, since these methodologies allow a greater response space and to fully extract all the possible data provided by each question.

## Figures and Tables

**Table 1:** Demographic characteristics.

	Gender Subgroup	
	Transgender (*n* = 155) *n* (%)	Non-binary (*n* = 59) *n* (%)
**Sexual Orientation**		
Heterosexual	73 (47.1)	2 (3.4)
Gay	5 (3.2)	1 (1.7)
Lesbian	3 (1.9)	9 (15.3)
Bisexual	17 (11)	7 (11.9)
Pansexual	49 (31.6)	29 (49.2)
Other	3 (1.9)	9 (15.3)
Missing	4 (2.6)	1 (1.7)
**Religion**		
Catholic	26 (16.8)	1 (1.7)
Atheist or agnostic	99 (63.9)	50 (84.7)
Other	12 (7.7)	6 (10.2)
Missing	18 (11.6)	2 (3.4)
**Area**		
Rural	33 (21.3)	2 (3.4)
Urban	120 (77.4)	56 (94.9)
Missing	2 (1.3)	1 (1.7)

**Table 2:** Main themes and respective codes regarding the question “In your own words, what was your main difficulty when you were transitioning?”.

Main difficulty while transitioning (27 missing; 9 does not apply)	**Absence of Difficulties** (6)		
	Individual-related difficulties	-Age (5) -Lack of financial resource (7) -Area of living (1) -Doubts about one’s sexual identity (5) -Lack of self-acceptance (5)	
	Family-related difficulties	-Lack of familial acceptance and support (41) -Family rejection (4)	
	Process-related difficulties	Law-related difficulties	-Law (3) -Changing to official documents (5) -Bureaucratic procedures (2)
		Medical-related difficulties	-Uninformed medical professionals (5) -Non-collaborative medical professionals (2)
			-Accessing hormonal treatment (1)
		-Lack of information (11) -Beginning the transition process (3) -Slowness of the process (9)	
	Gender Minority Stress related difficulties	-Traditional gender dichotomy (5) -Invisibility (4) -Lack of social acceptance (7) -Social rejection (10) -Discrimination (6) -Internalized transphobia (2) -Society (8) -External attitudes about transgenderism (8) -Coming out (22)	
	Other difficulties	-Everything (1)	
		-Nonexistent (1)	
		-Finding a job (1)	
		-Fear (9)	
		-Psychological symptoms (4)	

**Table 3:** Main themes and respective codes regarding the question “What is the main difficulty that you are facing nowadays?”.

Main actual difficulty (9 missing; 9 do not apply)	**Absence of Difficulties (6)**	
	Gender-identity related difficulties	- Fear of it being a phase (2) - Indecision concerning gender identity (5) - Indecision concerning sexual orientation (2) - Going to the bathroom as a transgender (3)
	Process-related difficulties	- The process itself (6) - Beginning the process (8) - Changing to official documents (15) - Uninformed professionals (1) - Length of the transition process (4) - Lack of time availability (2) - Financial problems (15)
	Sociodemographic characteristics related difficulties	- Age (3) - Religious beliefs (3) - Studies/Academic context (5) - Weight (1)
	Intimate relationships related difficulties	- Finding a partner (5) - Engaging in a relationship (1)
	Job related difficulties	- Finding a job (8) - Present/future work situation (7)
	Gender Minority Stress related difficulties	- External opinions (1) - Lack of acceptance (20) - Discrimination (10) - Isolation (2) - Lack of social integration (1) - Fear of rejection/Rejection (6) - Invisibility (9) - Having to justify one’s gender (2) - Social pressure to conform to the norm (2) - Misgendering (11)
	Psychological Symptoms and/or Disorders	- Gender dysphoria (17) - Depression (11) - Anxiety (9) - Eating disorders (1) - Self-esteem (1) - Solitude (1) - Lack of emotional control (1)

**Table 4:** Main themes and respective codes regarding the question “Do you think that the cause of this difficulty is your sexual orientation or your gender identity?”.

Cause of the main difficulty (13 missing; 1 does not apply)	Uncertain (8)		
	Sexual Orientation (SO; 2)		
	Gender identity (GI; 60)		
	Both sexual orientation and gender identity		Totally due to SO and GI (13) Partly due to SO and GI (13)
	Partly both		
	Other causes	Lack of acceptance (6) Lack of determination (1) Pathologization (1) Body image (1)	
		Gender Minority Stressors	Society (6) Transphobia (1) Discrimination (1) Rejection (1) Invisibility (1) Uniformed people concerning the non-binary (1)

**Table 5:** Main themes and respective codes regarding the question “In your own words, if you were capable of changing something right now, what would it be and why?”.

Things that participants would like to change (23 missing; 1 refused to answer)	Nothing (11)	
	Everything (2)	
	Personal Aspects	Age (3) Area of residence (6) Being afraid (3) Disconnection between body and emotional dimension (1) Being born (1)
	Biological Aspects	Voice (3) Physical appearance (24) Genitals (5) Having breasts (7) Changing the sex they were born with (7)
	Social Aspects	Familial acceptation and support (16) Friendships (4) Bullying victimization (1) LGBT community and services (1) Not having an intimate partner (1) Not having a support network (1)
	Financial aspects	Economic situation (9) Not being financially independent (8)
	Gender Minority Stress related aspects	Coming out of the closet (10) Society (12) Social inequalities (1)
	Transition Process related aspects	Changing official documents (8) Hormonal treatment (6) Nobody knowing one’s sex at birth (1)
	Gender related aspects	Being able to choose one’s gender (5) Being born transgender (21)
